# Acinar Cell Carcinoma of the Pancreas with Colon Involvement

**DOI:** 10.1155/2014/389425

**Published:** 2014-01-16

**Authors:** Naoki Asayama, Yasushi Kojima, Tomonori Aoki, Chiaki Maeyashiki, Chizu Yokoi, Mikio Yanase, Ryuichiro Suda, Hideaki Yano, Harumi Nakamura, Toru Igari

**Affiliations:** ^1^Department of Gastroenterology and Hepatology, National Center for Global Health and Medicine, 1-21-1 Toyama, Shinjuku-ku, Tokyo 162-8655, Japan; ^2^Department of Surgery, National Center for Global Health and Medicine, 1-21-1 Toyama, Shinjuku-ku, Tokyo 162-8655, Japan; ^3^Department of Pathology, National Center for Global Health and Medicine, 1-21-1 Toyama, Shinjuku-ku, Tokyo 162-8655, Japan

## Abstract

We report a case of acinar cell carcinoma of the pancreas with colon involvement that was difficult to distinguish from primary colon cancer. A 60-year-old man was admitted with a 1-month history of diarrhea. Contrast-enhanced computed tomography (CT) revealed a large tumor (10.6 × 11.6 cm) at the splenic flexure of the colon. Colonoscopy showed completely round ulcerative lesions, and biopsy revealed poorly differentiated adenocarcinoma. Left hemicolectomy, resection of the jejunum and pancreas body and tail, and splenectomy were performed based on a diagnosis of descending colon cancer (cT4N0M0, stage IIB), and surgery was considered to be curative. Diagnosis was subsequently confirmed as moderately differentiated acinar cell carcinoma of the pancreas by immunohistochemical staining (pT3N0M0, stage IIA). Multiple liver metastases with portal thrombosis were found 8 weeks postoperatively. Despite combination chemotherapy with oral S-1 and gemcitabine, the patient died of hepatic failure with no effect of chemotherapy 14 weeks postoperatively. Correct diagnosis was difficult to determine preoperatively from the clinical, CT, and colonoscopy findings. Moreover, the disease was extremely aggressive even after curative resection. Physicians should consider pancreatic cancer in the differential diagnosis of similar cases.

## 1. Introduction

Acinar cell carcinoma of the pancreas (ACC) is a rare malignant epithelial tumor representing 1-2% of all exocrine pancreatic neoplasms [1]. Symptoms such as weight loss, abdominal pain, nausea, and vomiting are nonspecific and are related mostly to either locally advanced tumors or metastasis [1, 2]. We present in this report a case of ACC with colon involvement that was difficult to distinguish from primary colon cancer.

## 2. Case Presentation

A 60-year-old man with no remarkable past history was admitted to our hospital in October 2011 with a 1-month history of diarrhea and nonspecific lower abdominal pain. He also complained of losing 25 kg within the previous 2 months. He did not regularly drink alcohol and had no history of acute pancreatitis or trauma. On admission, a hard mass, approximately 15 cm in size, with an irregular surface was palpable in the left abdomen. Initial laboratory tests revealed a white blood cell count of 23,960/*μ*L (normal: 3500–8500/*μ*L; 91% neutrophils; normal: 46–61%), a hemoglobin level of 5.8 g/dL (normal: 13.5–17.0 g/dL), and a platelet count of 32.0 × 10^4^/*μ*L (normal: 15–35 × 10^4^/*μ*L). Serum carbohydrate antigen 19.9 (CA19–9) was 48.9 U/L (normal: 0–36.0 U/L), carcinoembryonic antigen (CEA) was 45.1 ng/mL (normal: 0–4.9 ng/mL), Span-1 was 36.0 U/mL (normal: 0–30 U/mL), and soluble interleukin-2 receptor was (s-IL-2 R) measured as high as 1137 U/mL (normal: 188–570 U/mL). Serum aspirate aminotransferase, alanine aminotransferase, and amylase were within the normal range.

**Figure 1:**
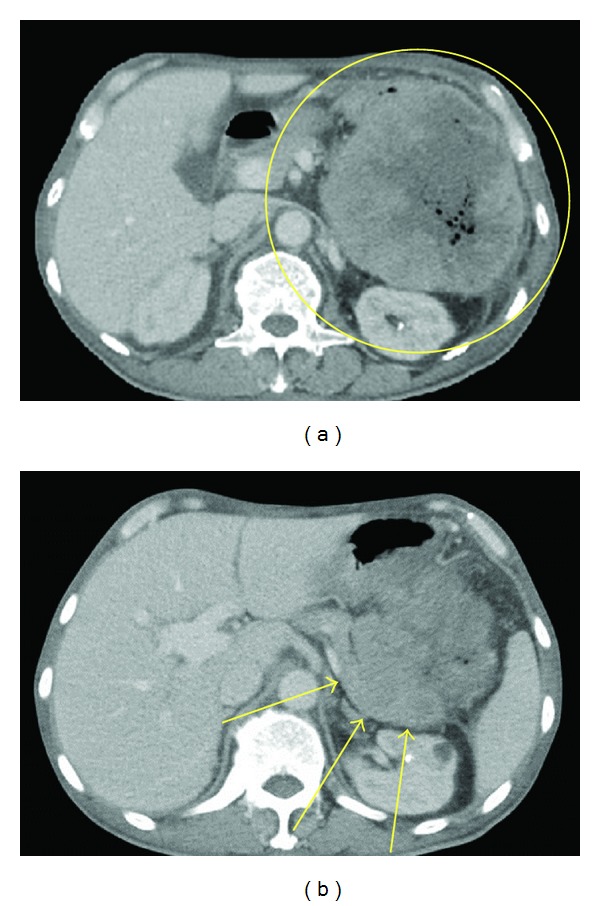
Preoperative abdominal computed tomography. (a) A large tumor (10.6 × 11.6 cm; enclosed by yellow circle) at the splenic flexure with an irregularly thickened wall and involving the tail of the pancreas; (b) tail of the pancreas (arrows).

**Figure 2:**
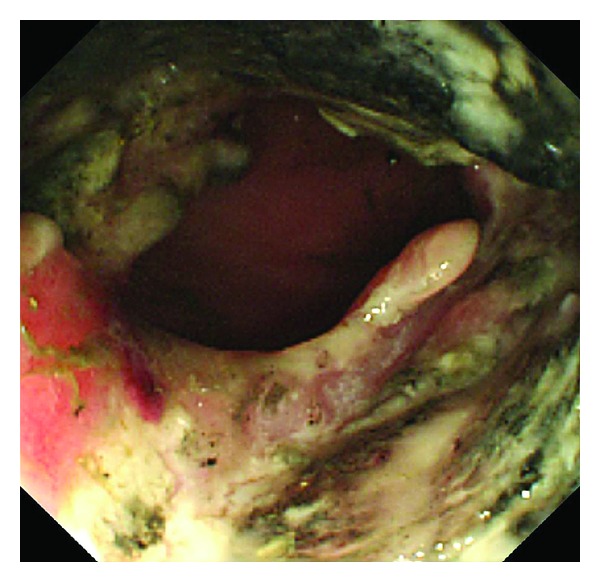
Colonoscopic findings of widely dispersed, completely round ulcerative lesions.

**Figure 3:**
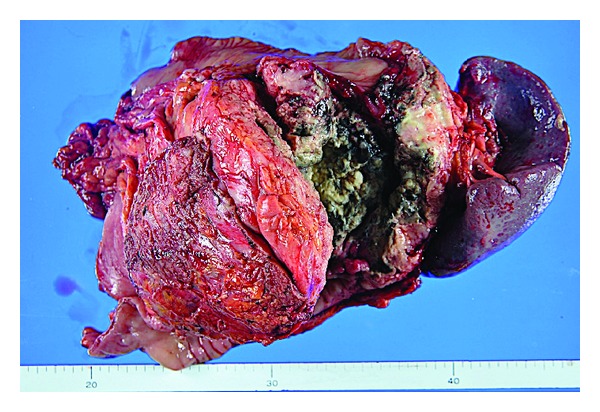
Macroscopic view of the resected tissue. In macroscopic view, the transverse colon is visible on the upper surface of the resected tissue, the large ulcer is visible at the front of the resected tissue, and the descending colon is visible on the lower surface of the resected tissue. The pancreas stump is not visible.

**Figure 4:**
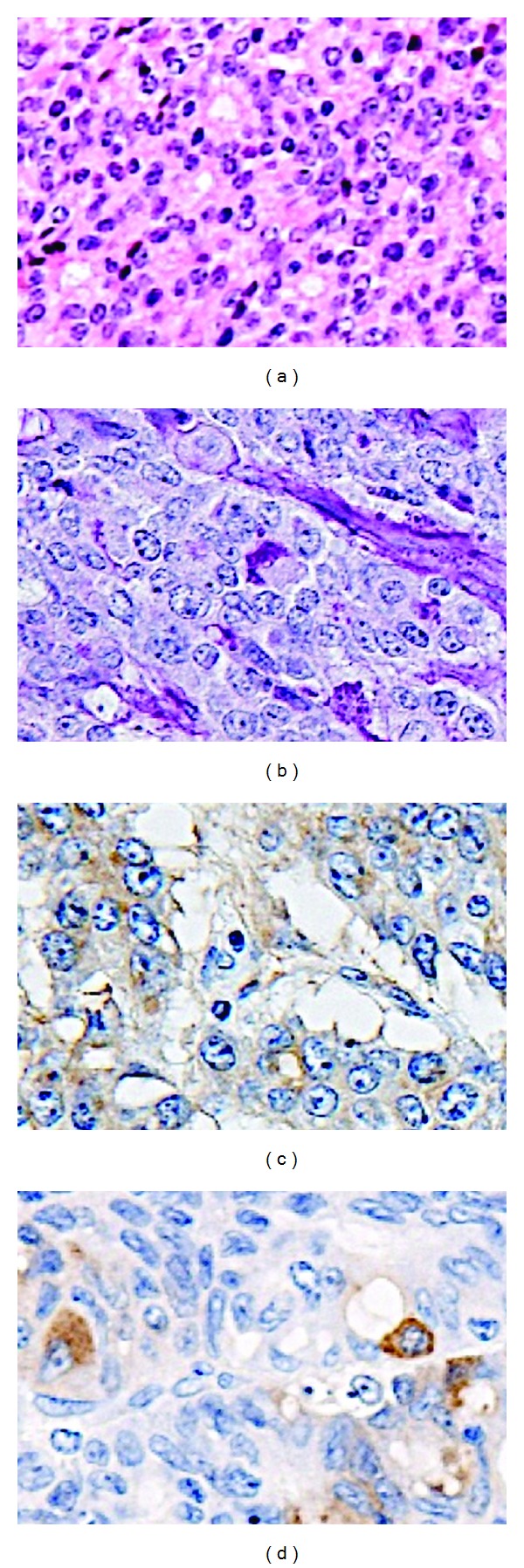
Microscopic findings of the resected tumor. (a) Acinar pattern consisting of well-formed acinar structures (hematoxylin and eosin staining, ×200); (b) a positive periodic acid-Schiff reaction followed diastase digestion within the cytoplasm and apical cytoplasmic tips (×200); tumor cells were focally positive for (c) lipase and (d) trypsin (×200).

**Figure 5:**
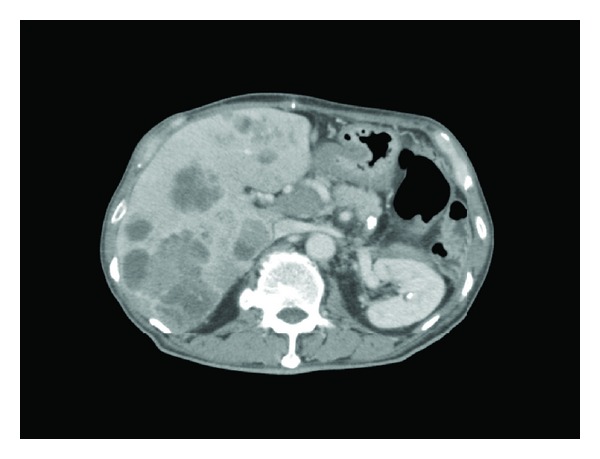
Abdominal computed tomography at the time of recurrence. Multiple liver metastases with portal thrombosis were found 8 weeks after surgery.

Contrast-enhanced computed tomography (CT) showed a large tumor (10.6 × 11.6 cm) at the splenic flexure of the colon with an irregularly thickened wall (Figure 1). Colonoscopy revealed completely round ulcerative lesions dispersed from the splenic flexure to the descending colon (Figure 2). The pathological findings of the biopsy specimen showed poorly differentiated adenocarcinoma. Except for this area, colonoscopy showed normal mucosa throughout the colon. Subsequent contrast-enhanced magnetic resonance imaging and positron emission tomography revealed no metastasis, including in the liver. Left hemicolectomy, resection of the jejunum, resection of the pancreas body and tail, and splenectomy were performed based on a diagnosis of descending colon cancer (cT4N0M0, stage IIB) in late October 2011. During surgery, the tumor was observed to invade the jejunum and pancreas without involving the superior mesenteric artery, superior mesenteric vein, or stomach. The surgical specimen consisted of an irregular mass of tissue, containing a portion of the left colon and the jejunum and the pancreas and the spleen (14 × 9.5 × 7.0 cm) (Figure 3). Curative resection was performed, but severe lymphatic and venous invasion was found pathologically. Histopathology revealed an acinar pattern consisting of cells growing in well-formed acini, and a solid pattern characterized by sheets and cords of cell separated by a thin fibrovascular stroma. A positive periodic acid-Schiff reaction was noted following diastase digestion in the cytoplasm and apical cytoplasmic tips. Immunohistochemically, the tumor cells were diffusely positive for pancytokeratin (AE1/AE3), focally positive for lipase and trypsin, and negative for cytokeratin 7, cytokeratin 20, CDX2, and endocrine markers such as chromogranin, synaptophysin, and CD56. From these morphological and immunohistochemical findings, a final diagnosis was made of moderately differentiated ACC with multiple organ (colon, small bowel, and spleen) involvement (pT3N0M0, stage IIA; Figure 4). No increase was noted in the levels of serum lipase, amylase, or elastase-1 after the operation. Despite an apparently curative resection, multiple liver metastases and portal thrombosis were found 8 weeks after surgery (Figure 5). Serum DUPAN-2 was 191 U/L (normal: 0–150 U/L). However, Serum CA19-9, CEA, Span-1, and alpha-fetoprotein (AFP) were within the normal range. Combination chemotherapy with oral S-1 (tegafur, 5-chloro-2,4-dihydroxypyridine, and oteracil potassium in a 1 : 0.4 : 1 molar ratio; 80 mg/body, days 1–14/q3w [3]) and gemcitabine (30 min intravenous injection of 1000 mg/m^2^ on days 1 and 8 of each 21-day cycle) was administered. However, the patient died of hepatic failure 14 weeks after surgery, with no effect of chemotherapy observed.

## 3. Discussion

Commonly referred clinical symptoms of ACC are upper abdominal pain, weight loss, nausea, and vomiting [1, 2]. In our case, it was diarrhea that led us to discover ACC as the neoplasm in the tail part of the pancreas, where it involved the colon expansively and formed a stricture. ACC often produces digestive enzymes such as trypsin, chymotrypsin, lipase, and amylase [1]. However, there were no increases in the levels of serum lipase, amylase, or elastase-1 in the present case, and we did not measure serum trypsin.

According to the initial symptoms of diarrhea, lower abdominal pain, and body weight loss, as well as the finding of the palpable mass in the left abdomen, colorectal cancer was considered the most probable diagnosis. In addition, CT showed a large, well-defined tumor mainly at the splenic flexure of the colon in contact with a part of the pancreas tail. Round ulcerative lesions of about 20 cm, detected by colonoscopy inside the colon, were an atypical finding of colon cancer. s-IL-2 R levels were as high as 1137 U/mL. We therefore suspected malignant lymphoma in addition to colon cancer, despite the fact that primary colonic lymphoma is rare [4], and we did not consider differential diagnosis of pancreatic tumor, including pancreatic cancer and solid pseudopapillary tumor. Based on the CT findings alone, solid pseudopapillary tumor of the pancreas would also have been a differential diagnosis because CT showed the patient's tumor as a large solid pseudopapillary tumor [5]. However, the patient was elderly and the tumor did not in fact have characteristic findings of a solid pseudopapillary tumor, such as calcification or a combination of solid and cystic components. We also considered that severe stenosis in the descending colon was the cause of diarrhea and that an operation was needed to avoid complete obstruction of the bowel. Surgery was subsequently performed based on a working diagnosis of descending colon cancer.

Gross examination revealed an annular constricting mass that appeared to be growing into the wall of the colon from the outside and pushing onto the mucosa, resulting in focal areas of ulceration. No heterotopic pancreatic tissue was seen in the colon. Tumor cells were negative for both CK20 and CDX2, specific markers of adenocarcinoma of intestinal origin. Taken together, moderately differentiated ACC with multiple organ involvement was diagnosed.

It is rare to find colon involvement in pancreatic cancer [6]. The most common sites of metastasis in ductal cell carcinoma are the liver (21–80%), lung (7–28%), abdomen (25%), regional lymph nodes (18–25%), and peritoneum (11–23%) [7]. Cases of ACC with colon involvement are also quite rare [8]. The metastatic rate of ACC at presentation is reported to be 30–50%, with the liver being the most common site [1, 2, 9]. In our patient, it was difficult to determine the correct diagnosis preoperatively on the basis of clinical symptoms and physical, CT, and colonoscopy findings. However, such findings may be indicative of ACC since this type of cancer is reported to develop from the parenchyma of the pancreas, show invasive exophytic growth with only mild changes in pancreatic tissue, and be large (on average, 10 cm in diameter) and circumscribed [1].

Reports from a few small case series have indicated that, compared with ductal cell carcinoma, ACC is considered to have good prognosis if completely resected [2, 10, 11]. A better prognosis can also be anticipated in patients younger than 60 years with a non-lipase-secreting tumor of <10 cm [1]. In another study, multivariable analysis revealed that age <65, well-differentiated tumor tissue, and negative resection margins were independent prognostic factors for acinar cell carcinoma [12]. We consider that our patient had extremely aggressive disease, even after what was deemed a curative resection, because he had worsened prognostic factors such as advanced age, large tumor size, and severe lymphatic and venous invasion histologically in the resected specimen.

Production of AFP has been reported as a possible marker of ACC [2]. In the present case, serum levels of CEA, CA19-9, Span-1, and AFP were all within the normal range when multiple liver metastases were found. Therefore, these markers, which are conventionally used as initial indicators of tumor, were ineffective in our patient.

No optimal strategy has yet been determined for patients with synchronous or recurrent liver metastasis from ACC. It has been reported that gemcitabine is not sufficiently efficacious against this cancer, despite its wide use as a standard agent for the treatment of pancreatic adenocarcinoma [13]. Some case studies have reported tumor shrinkage through the use of fluoropyrimidine-based treatment [2]. In our patient, however, combination chemotherapy with oral S-1 and gemcitabine had no therapeutic effect. This relatively rare case of ACC with colon involvement was difficult to distinguish from colon cancer preoperatively. Moreover, the disease was extremely aggressive even after curative resection and chemotherapy. Physicians should consider pancreatic cancer as a differential diagnosis in similar cases.

## References

[1] Klimstra DS, Heffess CS, Oertel JE, Rosai J (1992). Acinar cell carcinoma of the pancreas: a clinicopathologic study of 28 cases. *American Journal of Surgical Pathology*.

[2] Seth AK, Argani P, Campbell KA (2008). Acinar cell carcinoma of the pancreas: an institutional series of resected patients and review of the current literature. *Journal of Gastrointestinal Surgery*.

[3] Shirasaka T, Shimamato Y, Ohshimo H (1996). Development of a novel form of an oral 5-fluorouracil derivative (S-1) directed to the potentiation of the tumor selective cytotoxicity of 5-fluorouracil by two biochemical modulators. *Anti-Cancer Drugs*.

[4] Wong MTC, Eu KW (2006). Primary colorectal lymphomas. *Colorectal Disease*.

[5] Kobayashi T, Ozasa M, Miyashita K (2013). Large solid-pseudopapillary neoplasm of the pancreas with aberrant protein expression and mutation of *β*-catenin: a case report and literature review of the distribution of *β*-catenin mutation. *Internal Medicine*.

[6] Bellows C, Gage T, Stark M, McCarty C, Haque S (2009). Metastatic pancreatic carcinoma presenting as colon carcinoma. *Southern Medical Journal*.

[7] Raghavan D, Brecher ML, Johnson DH (2006). *Textbook of Uncommon Cancer*.

[8] Matsuyama T, Ogata S, Sugiura Y (2004). Acinar cell carcinoma of the pancreas eroding the pylorus and duodenal bulb. *Journal of Hepato-Biliary-Pancreatic Surgery*.

[9] Holen KD, Klimstra DS, Hummer A (2002). Clinical characteristics and outcomes from an institutional series of acinar cell carcinoma of the pancreas and related tumors. *Journal of Clinical Oncology*.

[10] Kitagami H, Kondo S, Hirano S, Kawakami H, Egawa S, Tanaka M (2007). Acinar cell carcinoma of the pancreas: clinical analysis of 115 patients from Pancreatic Cancer Registry of Japan Pancreas Society. *Pancreas*.

[11] Wisnoski NC, Townsend CM, Nealon WH, Freeman JL, Riall TS (2008). 672 patients with acinar cell carcinoma of the pancreas: a population-based comparison to pancreatic adenocarcinoma. *Surgery*.

[12] Schmidt CM, Matos JM, Bentrem DJ, Talamonti MS, Lillemoe KD, Bilimoria KY (2008). Acinar cell carcinoma of the pancreas in the united states: prognostic factors and comparison to ductal adenocarcinoma. *Journal of Gastrointestinal Surgery*.

[13] Seki Y, Okusaka T, Ikeda M, Morizane C, Ueno H (2009). Four cases of pancreatic acinar cell carcinoma treated with gemcitabine or S-1 as a single agent. *Japanese Journal of Clinical Oncology*.

